# Supplementary material for: Analysis of chi angle distributions in free amino acids via multiplet fitting of proton scalar couplings
Source: Magn Reson (Gott). 2024 Aug 19;5(2):103–20. doi: 10.5194/mr-5-103-2024 (PMC11570886; doi:10.5194/mr-5-103-2024)
Supplement: The supplement related to this article is available online at: https://doi.org/10.5194/mr-5-103-2024-supplement. [file mr-5-103-2024-supplement.zip › Syed2024/data/fit1d_fitnmr_output.tar.gz!/fit1d/trp/500/bmse000050_simulation_1_nmredata/1H/resonances_1d.pdf]

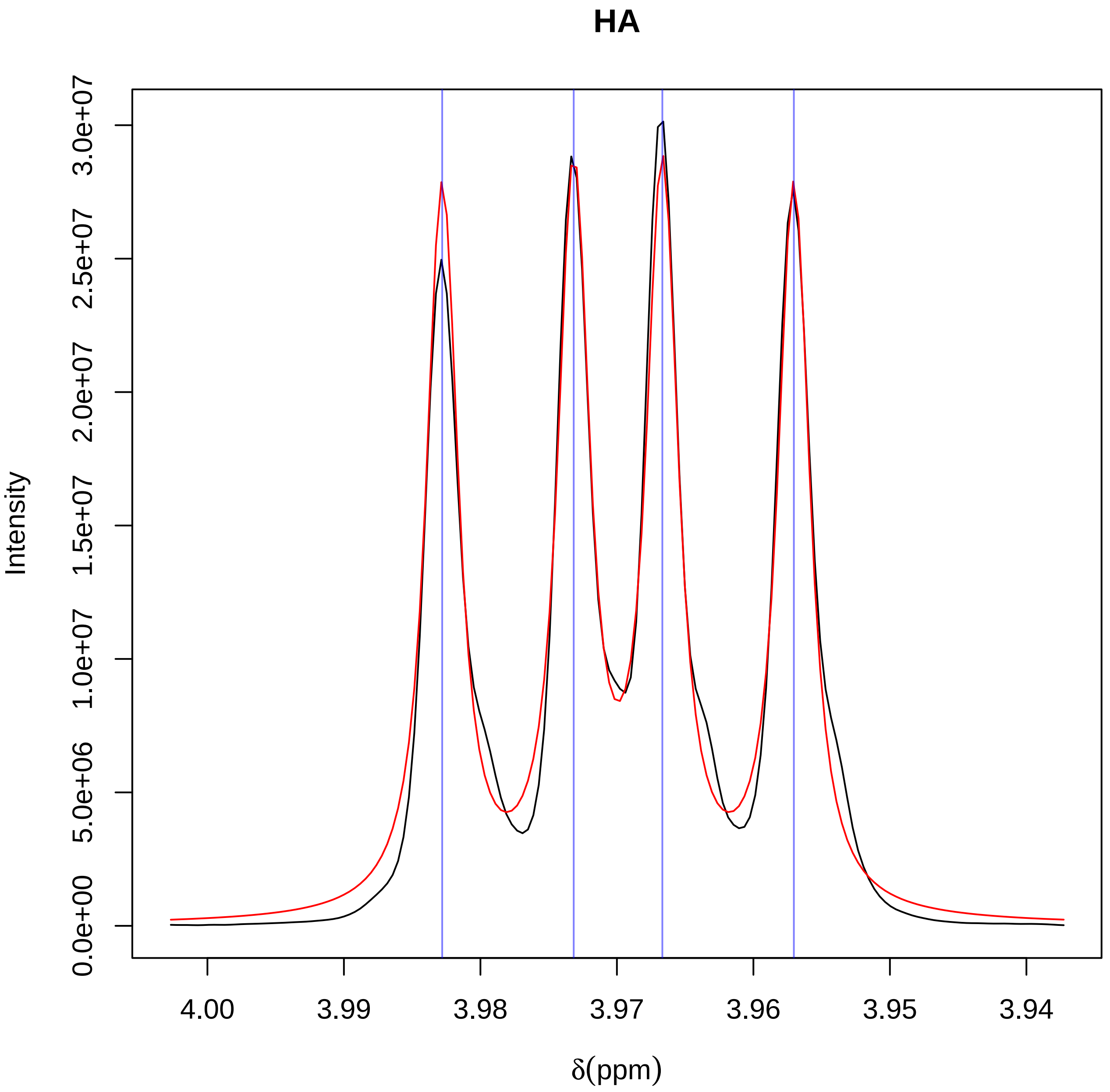

HA
Intensity
0.0e+00
5.0e+06
1.0e+07
1.5e+07
2.0e+07
2.5e+07
3.0e+07
4.00
3.99
3.98
3.97
3.96
3.95
3.94
δ(ppm)

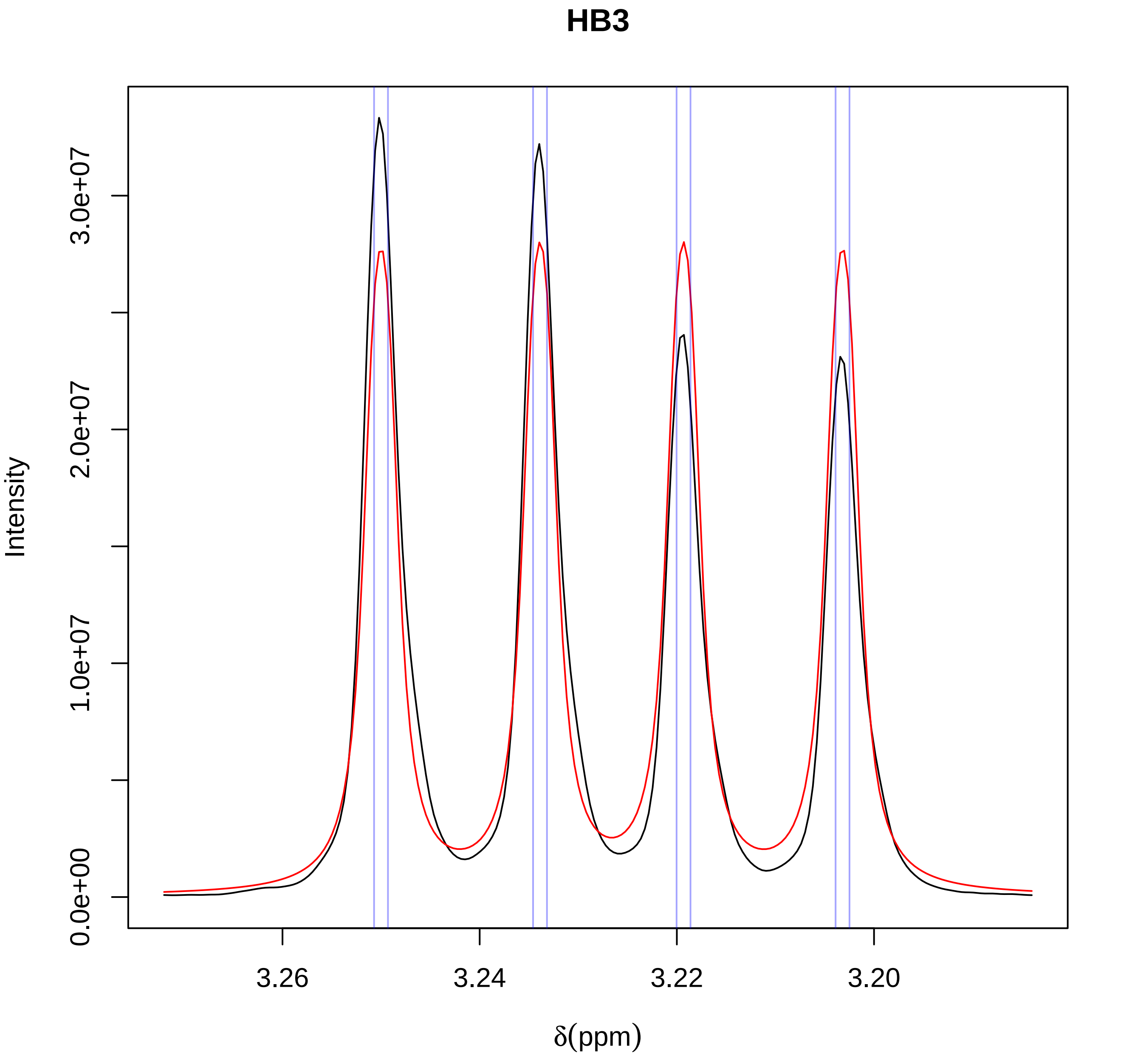

HB3
Intensity
0.0e+00
1.0e+07
2.0e+07
3.0e+07
3.26
3.24
3.22
3.20
δ(ppm)

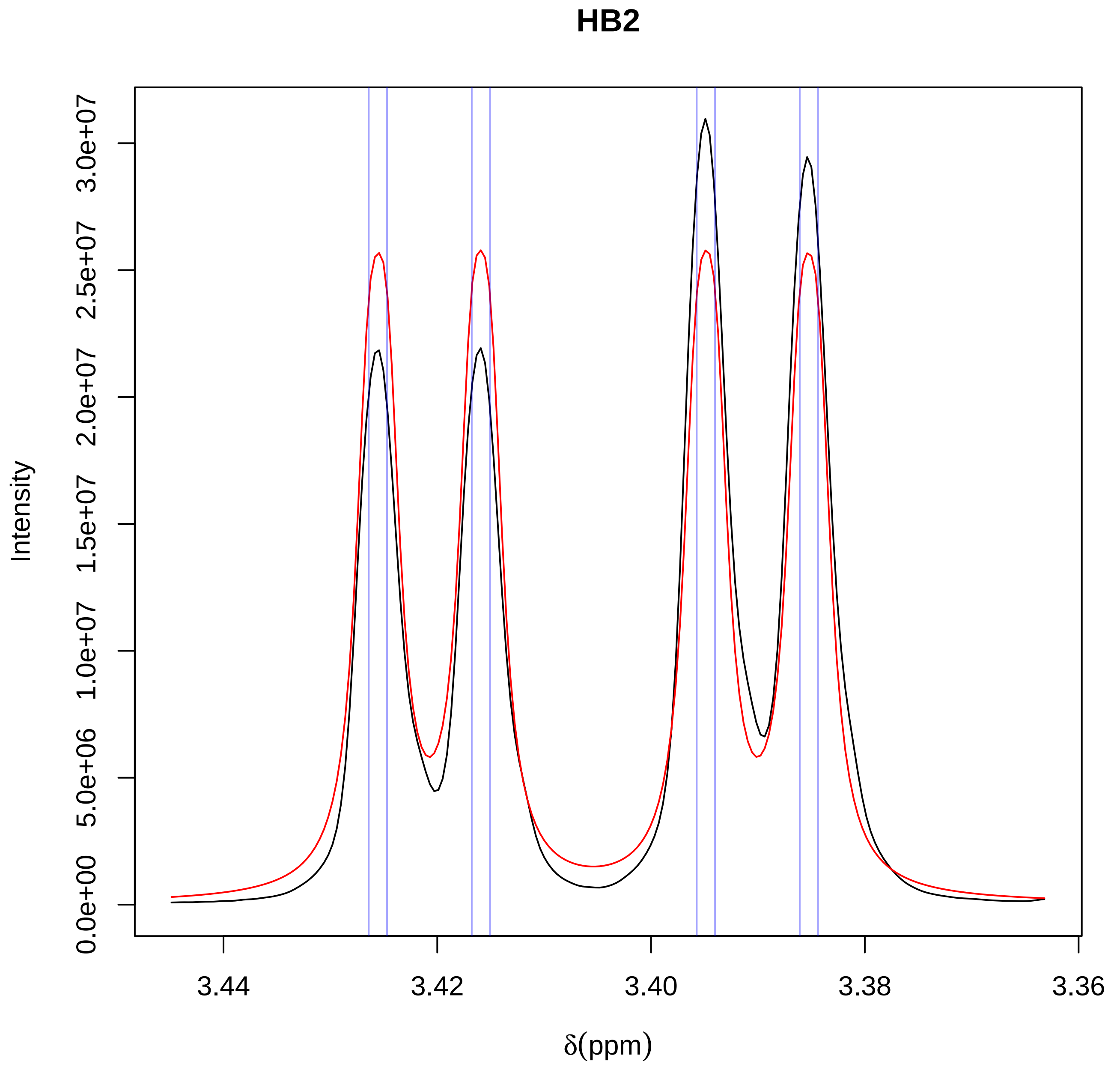

HB2
Intensity
0.0e+00
5.0e+06
1.0e+07
1.5e+07
2.0e+07
2.5e+07
3.0e+07
3.44
3.42
3.40
3.38
3.36
δ(ppm)

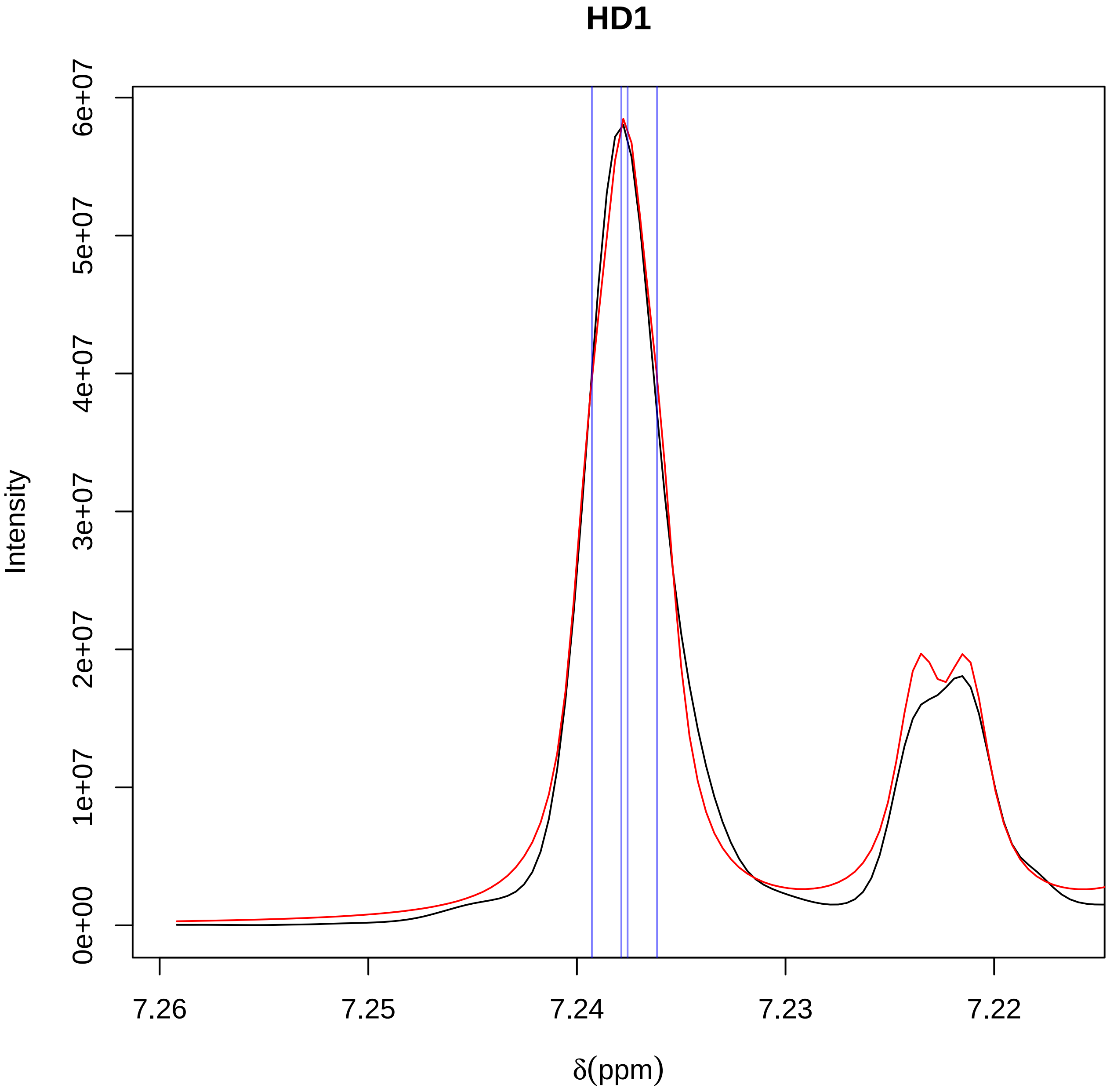
HD1
Intensity
0e+00
1e+07
2e+07
3e+07
4e+07
5e+07
6e+07
7.26
7.25
7.24
7.23
7.22
δ(ppm)

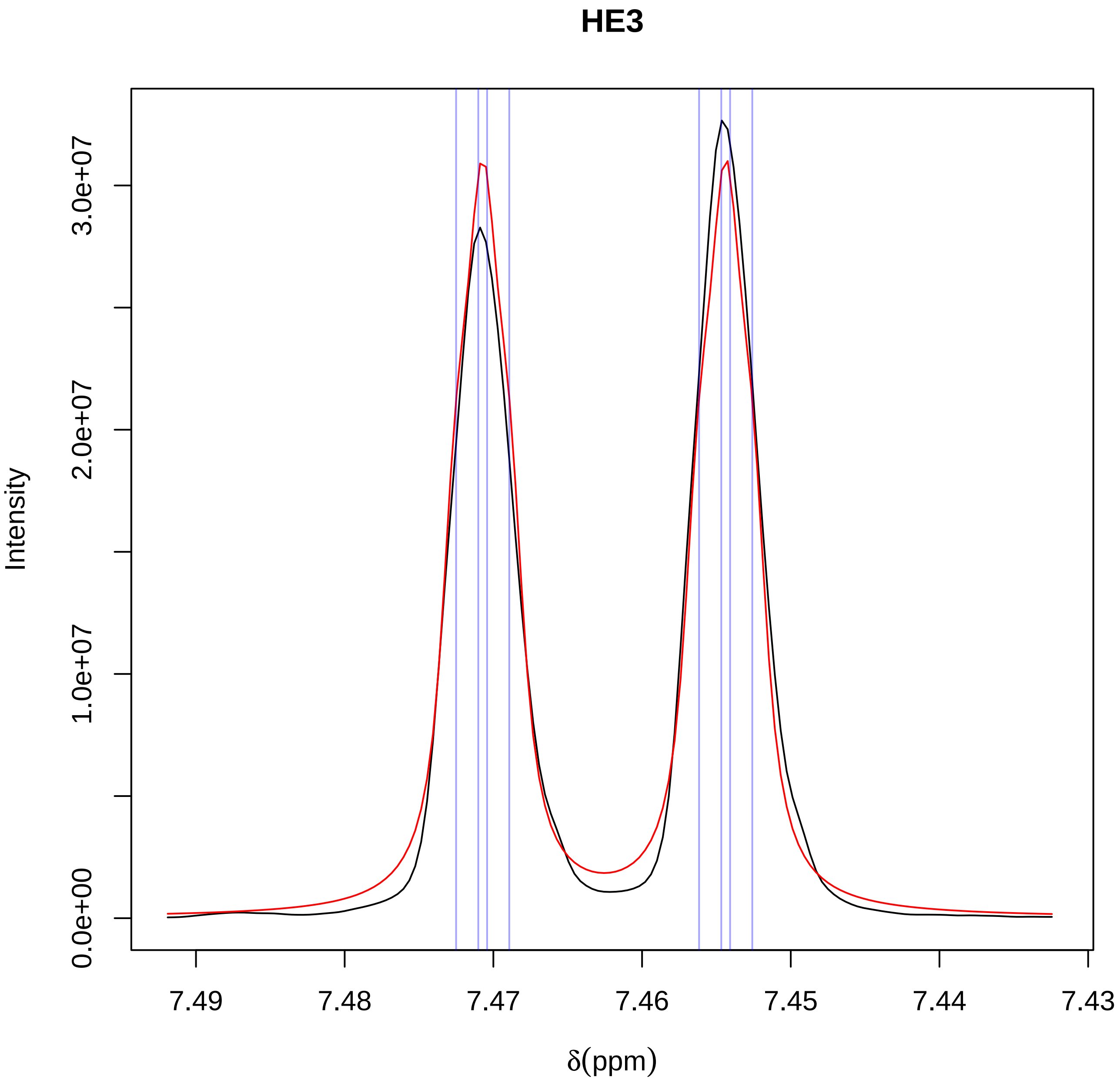
HE3
Intensity
0.0e+00
1.0e+07
2.0e+07
3.0e+07
7.49
7.48
7.47
7.46
7.45
7.44
7.43
δ(ppm)

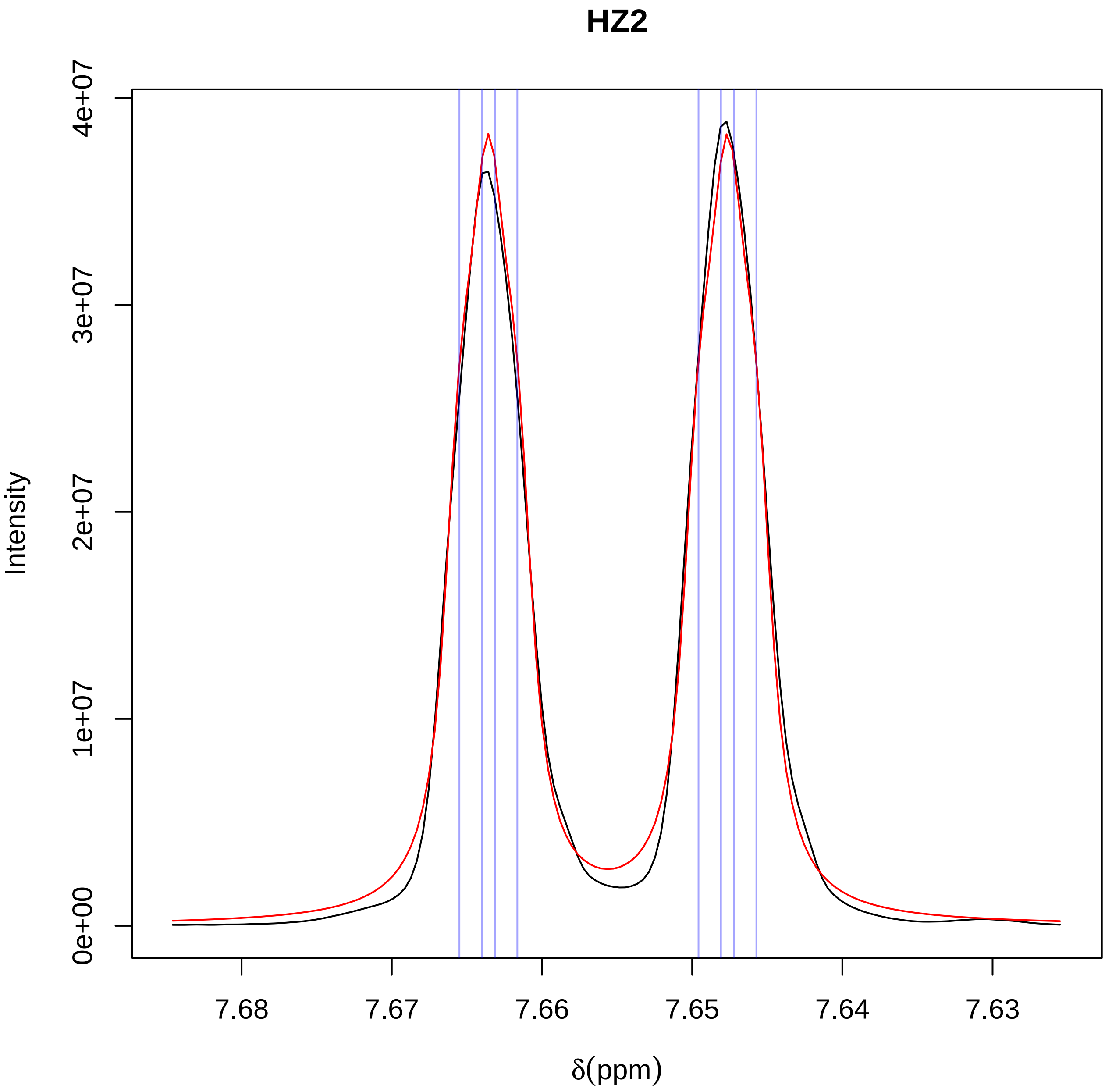

HZ2
Intensity
0e+00
1e+07
2e+07
3e+07
4e+07
7.68
7.67
7.66
7.65
7.64
7.63
δ(ppm)

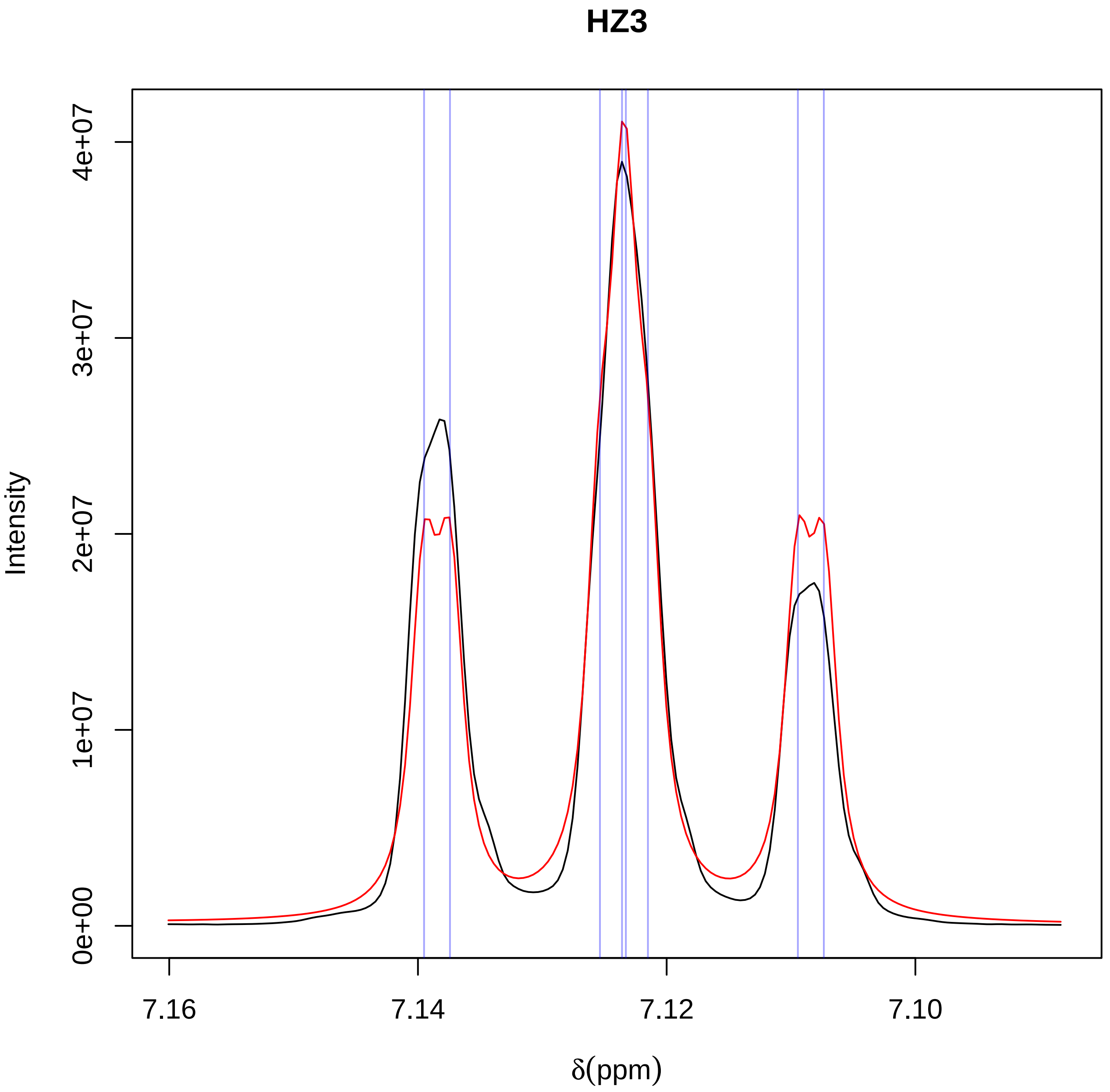
HZ3
Intensity
0e+00
1e+07
2e+07
3e+07
4e+07
7.16
7.14
7.12
7.10
δ(ppm)

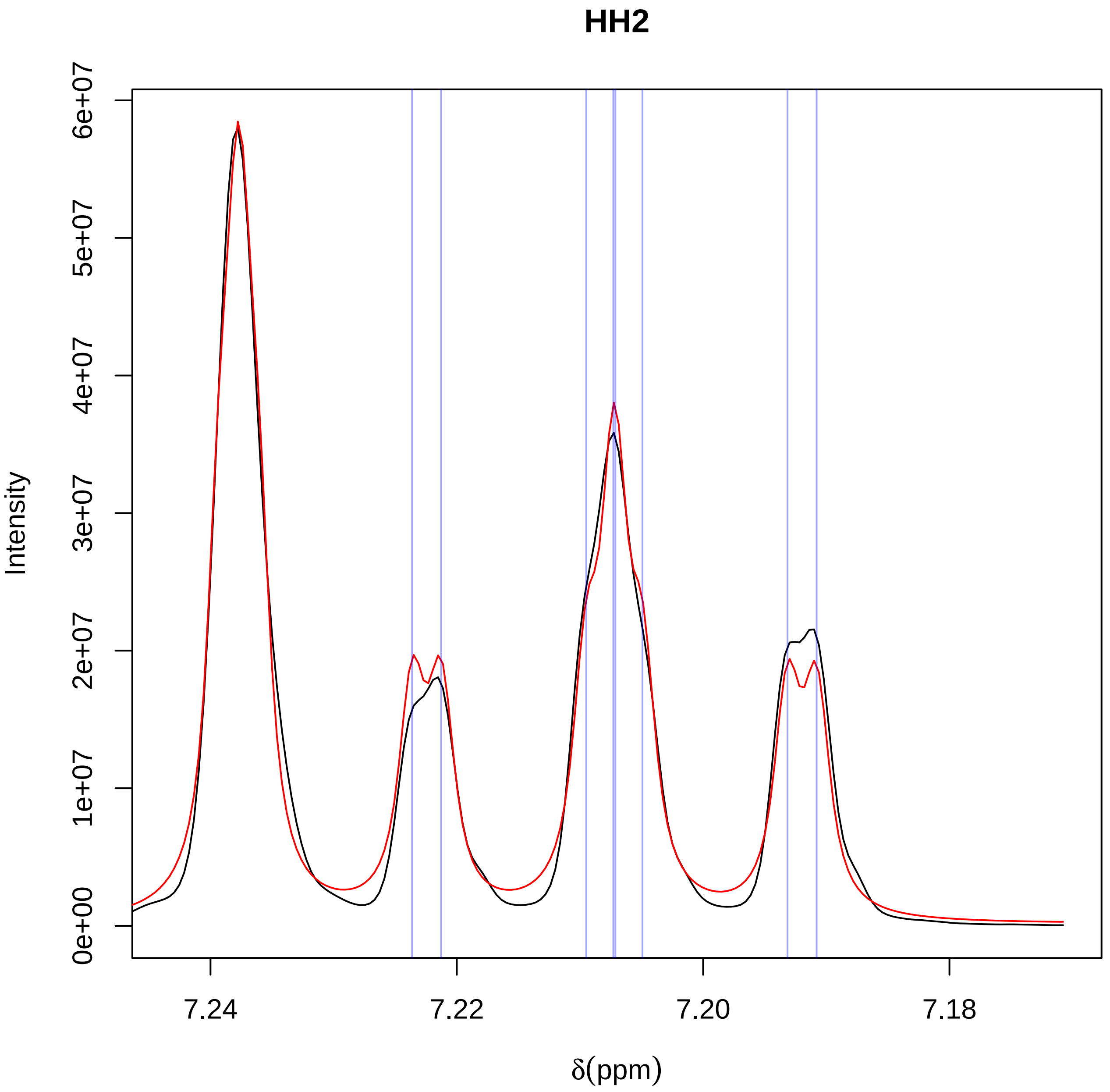
HH2
Intensity
0e+00
1e+07
2e+07
3e+07
4e+07
5e+07
6e+07
7.24
7.22
7.20
7.18
δ(ppm)
